# Exterior and Internal Uniform Loading of Pt Nanoparticles on Yolk-Shell La_2_O_3_ by Acoustic Levitation Synthesis with Enhanced Photocatalytic Performance

**DOI:** 10.3390/ma13010107

**Published:** 2019-12-25

**Authors:** Ming Qin, Qing Chang, Yinkai Yu, Hongjing Wu

**Affiliations:** 1 College of Chemistry and Chemical Engineering, Yan’an University, Yan’an 716000, China; qinming@mail.nwpu.edu.cn; 2 School of Physical Science and Technology, Northwestern Polytechnical University, Xi’an 710072, China; yyk@mail.nwpu.edu.cn

**Keywords:** Pt, La_2_O_3_, acoustic levitation, photocatalysis, synergistic effect

## Abstract

By the deposition of noble metal nanoparticles on a metal oxide substrate with a specific micro-/nanostructure, namely, yolk-shell structure, a remarkable improvement in photocatalytic performance can be achieved by the composites. Nevertheless, noble metal nanoparticles only distribute on the surface shell of metal oxide substrates when the conventional wet-chemistry reduction approach is employed. Herein, we proposed a novel acoustic levitation synthesis of Pt nanoparticles deposited on yolk-shell La_2_O_3_. The composites not only displayed well-defined, homogeneous distribution of Pt NPs on the exterior shell of La_2_O_3_ and the interior La_2_O_3_ core, but an enhanced chemical interaction between Pt and La_2_O_3_. The unique structure not only can display improved photocatalytic degradation rate toward methyl orange, but also may show great potential in fields of hydrogen generation, environmental protection, etc. The novel acoustic levitation synthesis can supplement the methodology of synthesizing well dispersed noble metal oxides over the whole yolk-shell structure through noble metal NPs deposition method.

## 1. Introduction 

Along with the rapid development of industry, dyestuff wastewater has become a severe environmental issue for their biodegradability and also carcinogenicity to human bodies [1,2]. Photocatalysis has been proven an effective way to eliminate dyes in wastewater by converting them into nontoxic matters without secondary pollution [3,4,5]. Composites composed of noble metal nanoparticles and metal oxides are promising photocatalysts due to the formation of Schottky barrier at the interface of these components, which can improve the photocatalytic performance by reducing the recombination of photogenerated charges [6,7]. Among the noble metals, Pt possess the highest work function, and thus is more suitable for the photocatalytic degradation of dyes [8,9,10]. In addition to the species of noble metals, the structures and morphologies of the substrate metal oxides also have significant influence on the photocatalytic behavior of the materials. Yolk-shell structure reveals high potential in photocatalytic field due to the shortened diffusion path for the reactants as well as the enhanced light harvesting capability aroused from the multiple reflection of incident light [11,12,13]. Therefore, deposition of Pt NPs on yolk-shell metal oxides can be a promising alternative for high-performance photocatalysts. 

Though tremendous efforts have been devoted to the synthesis of Pt NPs deposited yolk-shell metal oxides composites, the conventional wet-chemistry reduction approach can only construct yolk-shell composites with Pt coatings on the exterior shells of the metal oxides [14,15,16]. The inhomogeneous distribution of Pt in the whole yolk-shell structure is adverse to attaining catalysts with high photocatalytic activity due to the fewer catalytic active sites resulting from the declined interfacial and interaction areas between noble metal NPs and metal oxides [17,18,19,20]. The performance of catalysts can be further improved by uniform loading of nanoscale noble metal catalysts on metal oxide nanostructures. In spite of the uniform dispersion of Pt NPs on both of the exterior and interior surface of yolk-shell structure by one-step spray pyrolysis process having been reported [21,22,23], the research on uniformly depositing Pt NPs on both outside shell and inside core in yolk-shell metal oxides through the deposition method has not been reported. 

Herein, we proposed a novel acoustic levitation method to prepare uniform dispersion Pt NPs over the whole yolk-shell La_2_O_3_. La_2_O_3_ was chosen as the substrate for its excellent catalytic performance and abundance. Compared with the materials obtained from conventional wet-chemistry reduction approach, the acoustic levitation synthesis of Pt/La_2_O_3_ revealed higher dispersity as well as higher photocatalytic activity toward methyl orange. Our research supplements the synthesis of well-defined homogeneous noble metal NPs/yolk-shell metal oxides by noble metal NPs deposition.

## 2. Experimental

### 2.1. Synthesis

The yolk-shell La_2_O_3_ was first prepared based on our previous research [24,25]. Typically, D-glucose and lanthanum nitrate in a molar ratio of 1:1.85 were co-dissolved in deionized water to form a homogeneous solution. Then, the resultant mixture was transferred to a 100 mL capacity Teflon-lined stainless-steel autoclave and maintained at 180 °C for 20 h. Precursors were obtained by washing the precipitates with distilled water and ethanol. To harvest the final yolk-shell La_2_O_3_, precursors were calcined at 500 °C for 3 h. For the preparation of 1%-Pt/La_2_O_3_ composites by acoustic levitation approach, the synthesis process included three steps. Firstly, 0.1 g of the as-prepared yolk-shell La_2_O_3_ was added into 20 mL of 500 mg/L aqueous chloroplatinic acid solution. Then, 1% of polyvinyl alcohol (PVA) was dispersed into the above solution acting as the protecting agent, where the mass ratio between PVA and Pt was 3:2. After 30 min of stirring, the uniformly mixed precursor solution was obtained. Secondly, 100 μL of the abovementioned mixture was levitated via ultrasound. The acoustic levitator was custom built and comprised an emitter and reflector arranged coaxially along the gravitational direction [26]. To ensure the occurrence of the liquid drops, the equipment was worked at a fixed frequency of 30 kHz. We levitated the liquid sample via the acoustic radiation force exerted on the sample surface as a result of the nonlinear effect of ultrasound. Then, 0.05 mol/L of adequate sodium borohydride solution was injected into the mixture without contacting the levitated liquid. By repeating this procedure, the Pt loaded La_2_O_3_ was fabricated in a container-free atmosphere. Finally, the products (labeled as S1) were collected by filtration and washing to remove the residual NaBH_4_, and further, drying for 12 h at 80 °C. With respect to the 1%-Pt/La_2_O_3_ composite prepared by conventional wet-chemistry reduction method, the only difference was the second step. Alternatively, 0.05 mol/L of adequate sodium borohydride solution was added into the above mixed solution under vigorous stirring to ensure the completed reduction of Pt. The samples synthesized through this strategy were labeled as S2.

### 2.2. Materials Characterization

The morphology and the element distribution of the composites were observed by FEI Talos F200X transmission electron microscope (TEM) with accelerating voltage of 200 KV, which was equipped with an energy dispersive spectrometer (EDS). The chemical states of elements in S1 and S2 were measured by Shimadzu Kratos Axis Ultra DLD X-ray photoelectron spectroscopy (XPS) using a monochromatic Cu Kα X-ray source. The crystalline structure was tested on Rigaku Ultima IV X-Ray Diffraction (XRD) equipment with Cu Kα radiation (λ = 0.1540598 nm). The UV–Vis diffuse reflectance spectra of the as-obtained samples were obtained by using a UV-2550PC spectrophotometer equipped with a BaSO_4_ integrating sphere.

### 2.3. Photocatalytic Experiment

The photocatalytic experiments were carried out as follows: 40 mg of the as-prepared 1%-Pt/La_2_O_3_ samples was added into 250 mL of 10 mg/L methyl orange (MO) solution and then placed into dark under stirring for 30 min to reach the adsorption-desorption equilibrium. After that, the photocatalytic reaction started under the irradiation of mercury lamp (UV wavelength of 365 nm) as the simulated ultraviolet light source. A total of 4 mL of the dye solution was taken out at the reaction at times 0, 10, 30, 60, 90 and 120 min. To inspect the residual concentration, the solution was centrifuged and the supernatant was measured by UNICO UV-2802PC/PCS Ultraviolet-visible spectrophotometer (UV–Vis). The degradation rate can be calculated through the following equation:

(1) $$photodegradation\;rate\;\;(\%)\;=\;(c_{0}\;-\;c)/c_{0}\;\times \;100\;=\;(A_{0}\;-\;A)/A_{0}\;\times \;100$$

where *c*_0_ is the initial concentration of MO solution, i.e., 10 mg/L in our case; *c* represents the residual concentration of the dye solution; *A*_0_ and *A* are the absorbance of MO solution at 0 min and *t* min measured by UV–Vis, respectively.

## 3. Results and Discussion

**Figure 1:**
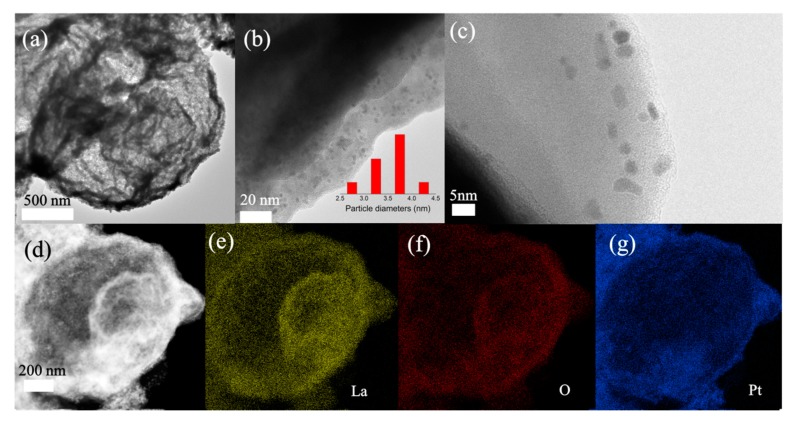
(**a**) TEM and (**b**,**c**) High-resolution TEM images of S1. (**d**) HAADF-STEM images of S2 and corresponding EDS elemental mapping images of (**e**) La, (**f**) O and (**g**) Pt.

According to our previous research, the hollow La_2_O_3_ microspheres comprised an outmost shell and an inner core. When employed as a photocatalyst, the cavity between the shell and core enable the material’s enhanced light use efficiency by providing multiple reflections and scattering. Thus, the well-defined yolk-shell La_2_O_3_ microspheres were selected as the metal oxide substrate due to their unique structure. The morphology of S2 remained a yolk-shell structure (Figure 1a) after the deposition of Pt NPs on the yolk-shell La_2_O_3_. The amplified TEM images demonstrate the Pt NPs were uniformly dispersed on the surface of the shell. Moreover, partial Pt NPs were embedded in into the oxides. The size distribution of Pt NPs ranged from 2.5 to 4.5 nm with an average grain diameter of 3.5 nm. The distribution of the elements in S2 was investigated and presented in Figure 1d–g. One can see the La and O elements are uniformly distributed over the yolk-shell structure. In contrast, the Pt only coats on the surface of the exterior shell without entering into the interior core. This inhomogeneous distribution of noble metals in yolk-shell structure could also be observed in previous research when the conventional wet-chemistry reduction approach was employed. 

**Figure 2:**
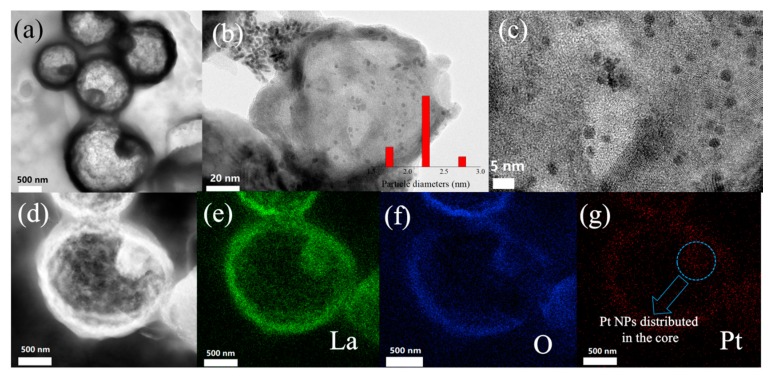
(**a**) TEM and (**b**,**c**) High-resolution TEM images of S1. (**d**) HAADF-STEM images of S1 and corresponding EDS elemental mapping images of (**e**) La, (**f**) O and (**g**) Pt, respectively.

Regarding the morphologies of S1 samples, the composites also retained the original yolk-shell structure after the acoustic levitation synthesis, yet they displayed distinct features compared with the S2 sample (Figure 2). Particularly, one can learn from the EDS mapping that the Pt in S1 reveals a peculiar distribution on the La_2_O_3_ substrate. Apparently, the Pt NPs not only coat on the exterior surface of La_2_O_3_, but the interior La_2_O_3_ core is wrapped by Pt NPs. In conventional synthetic process, the Pt NPs only disperse on the outmost shell of yolk-shell structure. As a result, the interfaces between the Pt NPs and La_2_O_3_ are greatly reduced, leading to the declined direct interfacial electronic transfer process between these components. In addition, the Pt NPs inside the core can be protected from aggregation by the La_2_O_3_ shell during the catalytic process, which is also benefit for a better photocatalytic activity. Simultaneously, based on our previous research [26], the Pt NPs obtained from acoustic levitation possess a smaller mean particle size of 2.3 nm. Also, numerous pores appear on the surface of La_2_O_3_ shell. The reduction in Pt NPs size can increase their specific catalytic activity [27,28] and the pores can effectively shorten the diffusion path for the reactants and charge carriers [29]. The abovementioned results demonstrate the significant role of acoustic levitation during the synthetic process. Therefore, the S1 sample is expected to display an enhanced photocatalytic performance than that of S2 sample.

**Figure 3:**
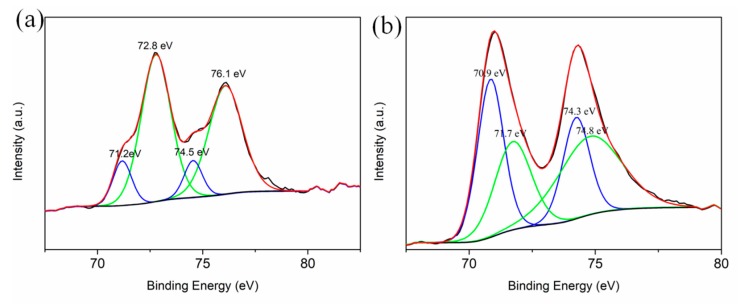
The XPS spectra of Pt 4f for (**a**) S1 and (**b**) S2.

The chemical states of the elements in S1 and S2 were investigated. Apart from the visualized physical contacts variation observed from EDS, the changing chemical contacts between Pt NPs and La_2_O_3_ substrate were disclosed by XPS measurement. The difference reflects the varied Pt valence state (the La, O and C elements are analogous and not presented here). For these samples, two peaks can be observed in Figure 3. After deconvolution, the peaks located at 71.2 eV and 74.5 eV correspond to Pt_7/2_ and Pt 4f_5/2_ with zero valence states [30]. The peaks around 72.8 eV and 76.1 eV are assigned to the Pt 4f_7/2_ and Pt 4f_5/2_, which are characteristic of the PtO_x_ phase originating from the Pt^δ+^ in the composites [31]. Obviously, the ratio of Pt^δ+^ in S1 sample is much higher than in S2 (in Table 1). Since the contacting area between Pt and La_2_O_3_ are enhanced by acoustic levitation synthesis, the interaction between the two components strengthened, and more La–O–Pt species formed on the surface. Thus, strengthened electronic metal-support interaction occurs at the Pt/La_2_O_3_ interface. This interaction is associated with charge transfer from Pt to the La_2_O_3_ support, resulting in the formation of more oxidized Pt^δ+^ species at the interface. Therefore, the novel acoustic levitation not only enlarges the physical contacts area between Pt NPs and La_2_O_3_ substrate, but reinforces the chemical interaction. Notably, strong interactions between the noble metal and the catalytically active metal oxide are maintained via physical or chemical contacts in the structure. In addition, it is reported that the Pt^δ+^ species in the composites are served as the active sites during the catalytic reaction [32]. Therefore, the variation in Pt states may also have a significant influence on the photocatalytic behavior.

**Figure 4:**
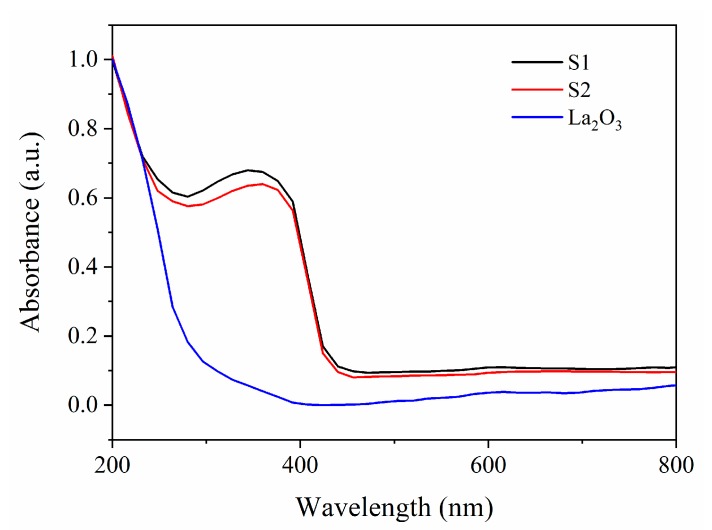
The UV–Vis diffuse reflectance spectra of La_2_O_3_, S1 and S2.

To inspect the light absorption for different samples, the UV–Vis diffuse reflectance spectra test was carried out (Figure 4). Compared with the bare La_2_O_3_ sample, both of S1 and S2 display improved light absorption capability. Additionally, the S1 reveals higher absorbance and blue shift than that of S2, which may originate from the unique structure of distribution characteristics of S1 as well as the strengthened chemical interaction between Pt NPs and La_2_O_3_ substrate. The absorption edges for these samples are in the UV light region; thus, the photodegradation of MO was accomplished under the irradiation of UV light.

**Figure 5:**
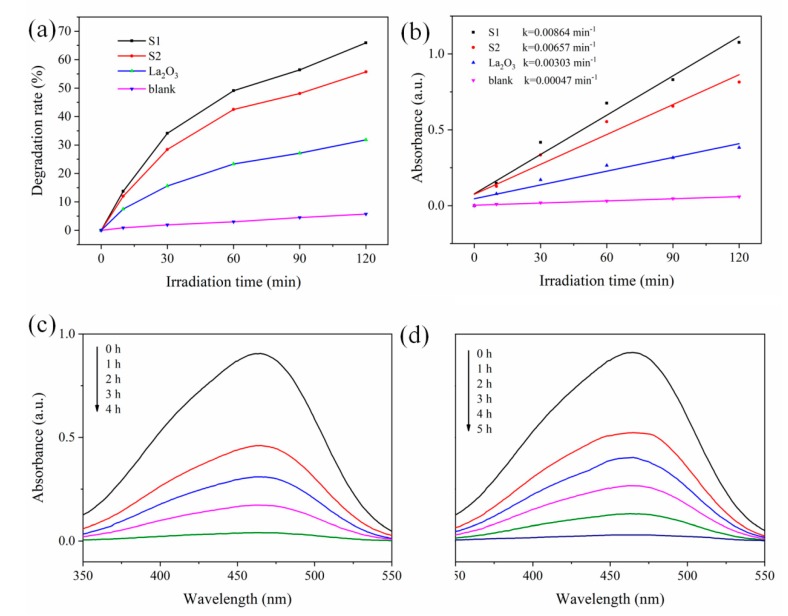
(**a**) Photocatalytic degradation of MO over the samples and (**b**) corresponding pseudo-first-order kinetic plots. The absorbance intensity–wavelength spectra of the variant composites with different times for (**c**) S1 and (**d**) S2.

The degradation rate for MO of the samples is shown in Figure 5. Without addition of catalysts, only 5% of MO degraded, indicating the MO is rather stable under UV light. For the pristine La_2_O_3_, 32% of MO degraded within 120 min. The degradation rate is remarkably enhanced for S1 and S2 at the same reaction time. This result verifies the synergetic catalytic properties by the coupling of Pt and La_2_O_3_. As expected, the S1 even displays a 10% higher degradation rate than the S2. Moreover, the MO can almost be removed within 4 h by S1, while it takes 5 h for S2. The pseudo-first-order rate constant of S1 reached 0.00864 min^−1^, the highest among the samples. These results confirm S1 displays much higher photocatalytic activity than S2, indicating that the Pt/La_2_O_3_ photocatalyst prepared by acoustic levitation approach can be a better alternative than that of produced by conventional wet-chemistry reduction method. As aforementioned, the superior photocatalytic performance originates from the uniform distribution of Pt NPs over the whole La_2_O_3_ yolk-shell structure; thus, it enlarges the interaction area and strengthens the synergetic catalytic effect. Meanwhile, smaller Pt NPs particle size as well as the appearance of pores on the surface of La_2_O_3_ shell also has a positive effect on the catalytic activity. 

It is common that the Pt NPs are only deposited on the exterior shell when regular synthetic strategies are employed to design noble metal deposited on yolk-shell metal oxides. However, it has been proven by our research that the inhomogeneous distribution of Pt NPs will suffer from the reduced interaction area between noble metal and metal oxides, resulting in a weakened synergetic effect. Though uniformly distributed noble metals over the whole yolk-shell metal oxides have been reported, the method takes advantage of the noble metal salts solution and metal salts simultaneously, as the reactants and the yolk-shell structure are acquired through one-step spray pyrolysis process [21,22,23]. Research on synthesis of well-defined homogeneous noble metal NPs/yolk-shell metal oxides through deposition has not been reported as of yet. The formation of unique structure in our research is ascribed to the acoustic levitation technique. Firstly, the acoustic levitation provides a container-free synthesis condition, which avoids the collision between Pt nucleation and vessel wall. Consequently, the Pt species can more uniformly distribute on the reaction solution under acoustic stirring compared with the conventional approach. Secondly, abundant pores formed on the shell of La_2_O_3_ shell under the acoustic levitation process. The pores provide accesses for the Pt nucleation to enter the interior and deposited on the surface of inner core. By the more uniformly distributed Pt NPs and the relief of aggregation caused by collision, the mean particle size of Pt NPs decreases. Put simply, the acoustic levitation technique provides us a novel strategy for the preparation of well-defined homogeneous noble metal NPs/yolk-shell metal oxides. The unique structure not only can display improved photocatalytic performance, but also may show great potential in fields of hydrogen generation, environmental protection, etc. The novel acoustic levitation synthesis can supplement the methodology of synthesizing well-dispersed noble metal oxides over the whole yolk-shell structure through noble metal NPs deposition method. 

## 4. Conclusions

In this work, a Pt deposited yolk-shell La_2_O_3_ composite was successfully prepared by a novel acoustic levitation approach. In contrast to the inhomogeneous distribution of Pt on the outmost shell of the yolk-shell La_2_O_3_, the Pt NPs on samples produced through acoustic levitation possess higher dispersity and deposition on both outside shell and inside core in yolk-shell La_2_O_3_. The uniform distribution of Pt NPs can enhance the area in contact with the La_2_O_3_ substrate, and thus strengthen the synergistic effect between the two components. Consequently, improved photocatalytic performance was achieved by this unique structure. The peculiar structure also shows great potential in fields of hydrogen generation, environmental protection, etc. Our research proves the acoustic levitation method can be a reasonable supplement for the synthesis of well-defined homogeneous noble metal NPs/yolk-shell metal oxides by noble metal NPs deposition.

## Figures and Tables

**Table 1:** The Pt valence state distribution over the samples.

Samples	Pt with Zero Valence State (%)	Pt with Oxidation State (%)
S1	28.37	71.63
S2	56.25	43.75
